# Feasibility and Acceptability of an Overdose Prevention Intervention Delivered by Community Pharmacists for Patients Prescribed Opioids for Chronic Non-Cancer Pain

**DOI:** 10.3390/pharmacy11030088

**Published:** 2023-05-22

**Authors:** Joe Schofield, Tessa Parkes, Fiona Mercer, Rebecca Foster, Kristina Hnízdilová, Catriona Matheson, Wez Steele, Andrew McAuley, Fiona Raeburn, Lucy Skea, Alexander Baldacchino

**Affiliations:** 1 Salvation Army Centre for Addiction Services and Research, Faculty of Social Sciences, University of Stirling, Stirling FK9 4LA, UK; 2 NHS Lanarkshire, Bothwell G71 8BB, UK; 3 School of Applied Sciences, Edinburgh Napier University, Edinburgh EH11 4BN, UK; 4 School of Medicine, Molecular and Clinical Medicine, University of Dundee, Dundee DD1 4HN, UK; 5 Faculty of Social Sciences, University of Stirling, Stirling FK9 4LA, UK; 6 Independent Researcher, Edinburgh EH17, UK; 7 School of Health and Life Sciences, Glasgow Caledonian University, Glasgow G4 0BA, UK; 8 NHS Grampian, Aberdeen AB15 6RE, UK; 9 School of Medicine, St. Andrews University, St. Andrews KY16 9TF, UK

**Keywords:** community pharmacists, opioid overdose risk, chronic non-cancer pain, prescription opioids, overdose prevention, overdose intervention, naloxone

## Abstract

There have been increases in prescriptions of high strength opioids for chronic non-cancer pain (CNCP), but CNCP patients perceive themselves as being at low risk of opioid overdose and generally have limited overdose awareness. This study examined how an overdose prevention intervention (opioid safety education, naloxone training, and take-home naloxone (THN)) delivered by community pharmacists for patients prescribed high-strength opioids for CNCP would work in practice in Scotland. Twelve patients received the intervention. CNCP patients and Community Pharmacists were interviewed about their experiences of the intervention and perceptions of its acceptability and feasibility. CNCP patients did not initially perceive themselves as being at risk of overdose but, through the intervention, developed insight into opioid-related risk and the value of naloxone. Pharmacists also identified patients’ low risk perceptions and low overdose awareness. While pharmacists had positive attitudes towards the intervention, they outlined challenges in delivering it under time and resource pressures and during the COVID-19 pandemic. Overdose prevention interventions are required in the CNCP population as this group has elevated risk factors for overdose but are commonly overlooked. Customised overdose prevention interventions for CNCP patients attend to gaps in overdose awareness and risk perceptions in this population.

## 1. Introduction

Chronic pain is long-term, persistent pain that lasts more than three months [1]. Chronic pain affects more than 30% of people worldwide and is a leading cause of disease and personal burden [2]. UK estimates are even higher, with between one third and one half of the population experiencing chronic pain across their lifetime. Chronic pain impacts on physical health and is linked to a disruption of daily activities, including sleep problems and loss of earnings [1]. Chronic pain can cause significant psychological and emotional impacts, such as low mood and reduced quality of life [3]. People experiencing chronic pain are vulnerable to problem substance use and social isolation which can further exacerbate their experience of pain [4]. People who are prescribed opioids for chronic pain can have increased prescription opioid overdose risk [5,6,7].

Although evidence suggests that long-term prescribing of opioids for chronic non-cancer pain (CNCP) is inferior to either non-opioid medication or non-medication strategies for improving pain or disability-related function [1], opioids continue to be a common component of pain management for people with chronic pain, including CNCP. Opioids may decrease pain and improve function only for a minority [8], and there are concerns over their longer-term efficacy and potential for harm [9]. Recent guidance for patients in the UK proposes that opioids should not be the first course of action for people living with chronic pain [1]. Instead, non-pharmacological treatments, such as group exercise programmes or psychological therapy, should be offered first to help manage pain [1]. Opioids are associated with central nervous system depression, dependence, and possibly an increase in all-cause mortality [10,11,12]. In the UK, the Medications and Healthcare products Regulatory Agency now recommend that overdose risk should be communicated to all patients prescribed opioids for chronic pain [13].

Patients prescribed long-term opioids for chronic pain can experience significant socioeconomic disadvantage through their pain, impeding their ability to work [14]. Additionally, patients prescribed long-term opioids tend to have concurrent risk factors for non-prescription drug use, including experiencing chronic pain and poorer mental health [15,16]. While initiation of non-prescription drug use amongst patients prescribed opioids for CNCP is generally low, Wilton et al. outlined that this was still eight times higher than people who were ‘opioid naive’ [16]. The authors proposed that patients prescribed opioids for pain could transition to non-prescription drugs if their prescribed opioids were involuntarily tapered, abruptly stopped, and/or their pain was under-treated [16]. Coffin et al. also identified that reduced access to prescribed opioids amongst patients previously reliant on their prescription was related to an increased use of non-prescription opioid analgesia [17]. In the CNCP population, polypharmacy (the use of multiple medications at the same time, including the co-prescribing of other central nervous system depressants), high dose opioids (≥50 morphine milligram equivalent per day), histories of non-prescription substance use, and co-morbid mental and physical health conditions are all risk factors for opioid-related harms, including overdose [6,10,18]. Additionally, CNCP is over-represented in people who use non-prescription substances [19,20], and this has also been found in the Scottish context [21].

Although opioids are not the first recommended course of pain management for CNCP [1], prescribing trends for CNCP show an increase in the use of strong opioids, especially in high income countries [11]. Whilst recent data from North America has indicated a decrease in prescribing trends since 2016 [22], rates in Scotland have increased. This rise has not been equally distributed across the country. People from areas with higher levels of socio-economic deprivation are 3.5 times more likely to be prescribed a strong opioid when compared to those in the least deprived areas [23]. Scotland currently has the highest levels of drug-related deaths (DRDs) in Europe, and people living in more deprived areas are 18 times as likely to die from a DRD, compared to those living in more affluent areas [23]. Increases in opioid prescribing have coincided with increases in DRDs, and prescription and/or non-prescription opioids were implicated in 89% of DRDs in 2020 [23]. Current practices of recording DRDs in Scotland mean that prescription only DRDs cannot be distinguished from non-prescription DRDs, as all DRDs are registered without this distinction.

Whilst the risk and harms associated with the consumption of counterfeit/non-prescription drugs cannot be underestimated, the focus of the current paper is on the risks and harms associated with prescription opioid use. Supplying naloxone to people at-risk of overdose for peer administration—‘Take-Home Naloxone’ (THN)—was first implemented in Scotland in 2011 and delivered mainly via community drug services and to people released from prison [24]. In response to the recent rapid rise in opioid-related overdoses, there has been expansion of THN distribution outlets in Scotland, including via the ambulance service at the scene of an overdose, and through a free online ordering and delivery service managed by a drug and alcohol family support organisation. Whilst naloxone is available through the national THN programme to anyone at risk of, or likely to witness, opioid overdose, it is not widely distributed within the CNCP population [25]. Evidence suggests that naloxone programmes should make enhanced use of pharmacy networks to increase availability and accessibility of naloxone, given that pharmacists have regular contact with people who use opioids [26,27]. Additionally, the Royal Pharmaceutical Society recently discussed the role of pharmacies in reducing harm and preventing DRDs. Their first recommendation was that naloxone should be available from all pharmacies, with pharmacy staff receiving training to use it [28].

This feasibility study builds on findings from the Prescription Opioid Overdose Risk 1 study (described in previous papers by our team [6,29]), based in and funded by the Scottish National Health Service (NHS) Board NHS Fife. The study ran between 2019 and 2020 and analysed GP practice data to characterise patients at opioid overdose risk in Fife. Findings identified that, in the six months preceding the analysis, 42,382 patients were prescribed any opioid and, of these, 14,079 (33%) were prescribed a strong opioid. Additionally, considerable levels of comorbidities and polypharmacy that could increase the risk of harm were identified among this group [6].

Based on findings from the Prescription Opioid Overdose Risk 1 study, the study team developed a customised community pharmacy overdose prevention intervention for the CNCP population. Materials used in the current project built on resources from a similar intervention by Volpe et al. [30]. The team developed the intervention for CNCP patients at risk of prescription opioid overdose so that it could be delivered face-to-face in a pharmacy, or remotely using ‘Near Me’ software [31] to facilitate delivery under pandemic circumstances. The intervention development process has been described elsewhere [32]. The current study was designed to investigate how the intervention would work in practice, how acceptable it was to both CNCP patients receiving it and community pharmacists delivering it, and feasibility issues with its delivery in one Scottish Health Board area (NHS Grampian).

## 2. Materials and Methods

Community pharmacies in NHS Grampian were approached to participate in the study and invited to attend an online information and training session facilitated by the research team. This 1.5 h session was held online in early evening to reduce burdens on pharmacy staff during opening hours and was attended by ten pharmacists and technicians. Members of the study team presented an outline of the study aims and timescale; patient eligibility criteria and the recruitment process, and an overview of what was being asked of participating pharmacies. A discussion was facilitated to explore and address issues regarding delivery and how they could adapt their existing opioid and naloxone skills and knowledge to be more appropriate for CNCP patients.

Staff from those pharmacies that agreed to participate were sent a pack containing copies of all study materials, naloxone information, and details of relevant local and national services. Pharmacy staff involved in the study were required to complete the free Overdose Prevention, Intervention, and Naloxone online training module provided by the Scottish Drugs Forum. Twelve community pharmacies originally expressed interest in supporting the study. Of these, four were unable to confirm their participation and five withdrew early, mainly due to COVID-19 related pressures. In total, three pharmacies in NHS Grampian delivered the intervention to CNCP patients. Participating pharmacies were all independent (not part of larger national chains) and were located in a mix of city centre and rural town settings. All had received training and naloxone through their involvement in the NHS Grampian’s naloxone service for people who use drugs.

Eligible individuals had to be over 18 years old and be prescribed a strong opioid for CNCP for more than three months. The definition of strong opioids was guided by the research team’s clinical experts and advisors (DS, CM, AB) and by the British National Formulary [33]. Strong opioids were defined in the current study as medications containing diamorphine, fentanyl, hydromorphone, morphine, oxycodone, pentazocine, pethidine, tapentadol, tramadol, or buprenorphine patches. Total daily doses had to be equivalent to at least 50 mg of morphine. This study focused only on the CNCP population given that they are currently underserved in Scotland in terms of both research and overdose prevention interventions. For this reason, methadone, and buprenorphine (common Medication Assisted Treatments for substance dependency) were not eligible prescriptions: these patients could already access THN from participating pharmacies. Those previously prescribed THN were also excluded. We have used the term ‘patients’ in this study for those receiving the intervention given this was an NHS supported intervention.

**Figure 1:**
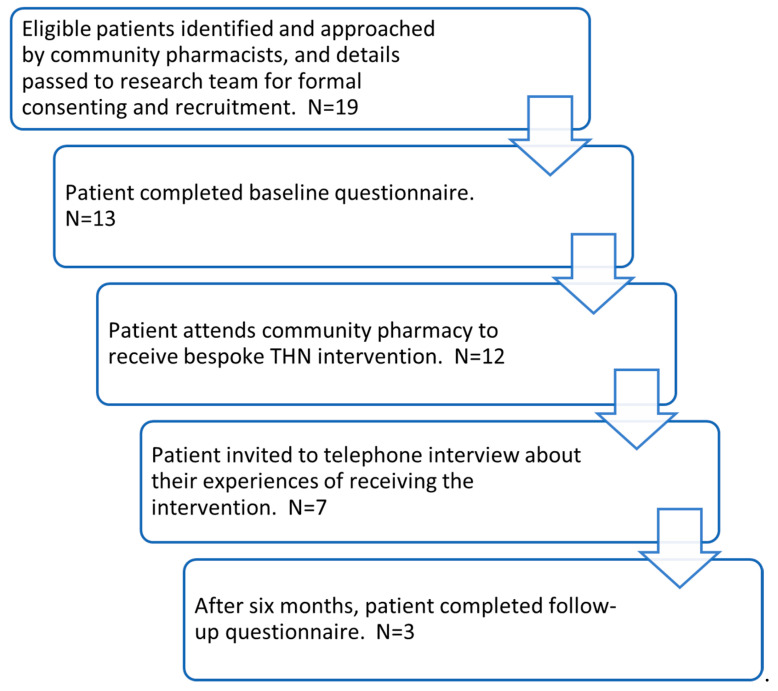
Study design and patient flow.

Patients were purposively recruited by community pharmacists based on the inclusion criteria. Some patients were approached based on existing pharmacy staff knowledge of those on high-dose opioid analgesics, and others identified when staff processed or dispensed an appropriate prescription. Contact details of eligible patients were passed on to the research team with the consent of patients. The research team then contacted the identified patients to discuss the intervention. If they were interested in taking part, participant information sheets and consent forms were sent and returned to the research team (Figure 1). Nineteen patients expressed interest in participating in the intervention and were sent a welcome letter. Of those, thirteen consented to participate and one subsequently withdrew from the study, leaving twelve patients who received the intervention. Not all patients who were at risk could be included, such as patients prescribed opioids for cancer pain, and this had implications for recruitment given that some pharmacies mainly dispensed opioids to patients with cancer pain.

This was a mixed methods study with quantitative (questionnaire) and qualitative (interview) data. The baseline questionnaire used checkboxes, multiple choice questions, and free-text fields to collect information on participants’ demographics, general health, prescribed medications, alcohol use, and other non-prescription drug use. The follow-up questionnaire repeated the non-demographic questions and additionally asked about participants’ perceptions and experience of the intervention via Likert-type scales and free-text fields. Both questionnaires included the six-item Prescription Opioid Overdose Misuse Index (POMI) to assess risk of opioid medication misuse [34]. Patients were either posted a paper copy of the questionnaire or emailed a link to an online version, depending on their preference.

Topic guides with open-ended questions were developed for patients (Supplementary File S1) and community pharmacy staff (Supplementary File S2). Seven patients took part in a telephone interview with a researcher to discuss their experiences of receiving the intervention. These lasted between 15 and 55 min and were audio recorded with consent. The shortest interview was with a participant with limited health literacy. All pharmacists who expressed interest in the intervention were also contacted for an interview. Both participating and non-participating pharmacists (those who wanted to take part but for a range of reasons were unable to) were invited to interview. In total, four pharmacists were interviewed, three who participated in the intervention, and one who was unable to be involved. Interviews lasted between 10 and 40 min and were audio recorded with consent.

After completing the baseline questionnaire, patients were invited to receive the intervention in-person at their community pharmacy or using ‘Near Me’ software for remote delivery. Despite this online option, all patients received the intervention in-person. Pharmacists delivered the intervention in the pharmacy consultation room. Intervention materials for patients included: an opioid safety card with basic information about opioid-related risk, naloxone use, and a safety plan to be used in the event of an overdose; a detailed information booklet, which expanded on information on the safety card, with instructions on when and how to use naloxone, and resources for further information and support; and a project website that had online versions of all intervention materials. The opioid safety card and booklet included materials adapted from a similar project conducted in Australia, with kind permission from Professor Suzanne Nielsen at Monash University [35]. Pharmacies received a study pack containing printed copies of all patient materials, an outline of the study, key points to cover and a sample script for patient recruitment, contact details for local specialist pharmacists (FR, LS) and members of the study team, and an outline of materials required and key points to cover when delivering the intervention to patients and any family members/carers who accompanied them.

The intervention included a discussion of opioid medication safety, a review of the patient materials, important messages to convey (patient safety, continuing to take pain medication as prescribed, contacting a healthcare professional if concerned, and local andnational information and support services), a demonstration on the use of naloxone, and time to check patient understanding and answer any questions. Naloxone (Prenoxad^®^ for intramuscular injection, or Nyxoid^®^ intranasal spray) was provided to patients.

A three-person Lived Experience Group, including people with CNCP and family members, was convened by WS to review all patient-facing intervention materials, questionnaires, and topic guides. The group met remotely and provided feedback on the study as it progressed, including suggesting changes for study materials and giving feedback on the final report and dissemination materials. Furthermore, a Research Advisory Group provided advice and guidance throughout the study; this group included clinicians and academics in the field, and the lead of a pain-specific voluntary sector service provider. Both groups were recruited via investigators’ existing contacts within Scottish pain management communities and other networks such as the Drugs Research Network for Scotland. Ethical approval was provided by the North of Scotland NHS Research Ethics Committee (REC) 1 which has jurisdiction for studies taking place in NHS Grampian [Project ID: 288945/REC Reference 21/NS/0014]. All experimental protocols were approved by the North of Scotland NHS REC 1 and the NHS Grampian Research and Development department. All methods were carried out in accordance with relevant guidelines and regulations. Informed consent was obtained from all study participants. To protect the anonymity of patients and pharmacists, no names were reported and some conditions that could identify patients were redacted. Alongside the interview quotes, gender (denoted as M for man and W for woman) and age (in years) are included to contextualise findings. Quotes from community pharmacists are pseudonymised as ‘pharmacist one’ etc. Approvals were granted to conduct this study during the COVID-19 pandemic with the understanding that it might have implications, such as reduced staff capacity in community pharmacies, which could impact the study, and which were detailed in the protocol risk assessment.

This was an intervention feasibility study and is part of the preparation work following the Medical Research Council’s complex intervention framework [36]. Descriptive statistics and figures were generated in Microsoft Excel (version 2203) by J.S. Interview data and were analysed to provide a thematic description of responses from patients and community pharmacists by F.M. and K.H., with guidance from T.P. Participants did not provide feedback on the findings but were invited to play an active role in the dissemination phase of the study. Initially, codes were generated inductively, but as codes became refined through the analysis process, they were also applied deductively to data. Themes were used to organise and map relationships between codes. Patient interviews were analysed separately from pharmacist interviews. Pharmacists are sometimes described as chemists in quotations.

## 3. Results

### 3.1. Patient Characteristics

**Figure 2:**
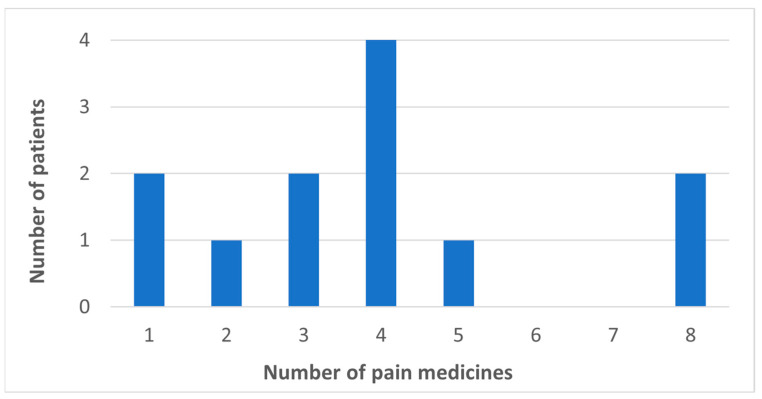
Number of pain medicines taken by patients.

All 12 identified as being of White ethnicity and three-quarters identified as male. Patients’ ages ranged from 33 to 74 years, with a median of 64 years. Two patients (17%) were taking one CNCP medication only (Figure 2). The median (IQR) number of concurrent pain medicines was four (2.75, 4.25).

In line with the inclusion criteria, all patients were prescribed at least one opioid. No patient reported use of other people’s medicines and/or non-prescription drugs (Table 1).

Four patients reported medication-related risk factors for opioid use disorder, and one person reported two risk factors (using medication more often and needing early refills) (Table 2). Self-report of two or more Prescription Opioid Misuse Index (POMI) risk factors is considered indicative of opioid misuse disorder [37].

Eleven patients reported a diagnosis of at least one comorbidity that could increase their risk of inappropriate use of/overdose from opioids (Table 3). Whilst Table 3 illustrates the comorbidities within the current sample, this may not be representative of the CNCP population.

Four patients had consumed alcohol in the previous month. Of these, two had consumed fewer than 14 units of alcohol (1 × 6, 1 × 8 units), and two drank more than 14 units (1 × 21, 1 × 30 units) per week. One patient reported using cannabis once a day on five days in a typical week.

Three patients completed the follow-up questionnaire six months after receiving the intervention (Table 4). They generally rated the written and in-person information as being easy to understand, helpful, and not upsetting. They thought the information was relevant to other patients on opioids but were more mixed in their views on its relevance to them. Whilst they learned about action to take on signs of overdose, they did not perceive themselves to be at risk of this.

### 3.2. Patient Interview Findings

All patients were invited to be interviewed after receiving the intervention. In total, seven patients participated. Three themes were developed from the qualitative analysis: relationship with health, medication, and overdose; experiences and perceptions of support; and perceptions of intervention and participation. Patients generally thought the intervention was valuable and important in developing their opioid overdose awareness. However, some patients initially perceived the intervention to be relevant only for people who used non-prescription drugs, or who intentionally ‘misused’ (a term used by some patients) drugs. However, this perception changed following the intervention, and patients all acknowledged that they were at some degree of opioid overdose risk.

#### 3.2.1. Theme One: Relationship with Health, Medication, and Overdose

Patient perceptions of the intervention were shaped by general health knowledge and wider health experiences. Overdose awareness was communicated by one patient through medication management strategies: for example, using a dosage box. The same patient acknowledged risk associated with their medication and identified a hesitancy to take more medication even when their pain increased: ‘*I mean it’s a real push before I take it. I don’t like taking any tablet that I don’t need to. […] if I can do without having an extra one for breakthrough pain, I would rather do without it*’ (M, 51). However, overdose awareness tended to be very low amongst those interviewed. Some patients identified that they did not know the signs of an overdose as they did not perceive themselves as being at risk:

*I didnae [didn’t] really know the signs and didnae think I needed to know*.

(W, 67)

For many patients, it was only through receiving the intervention that they became informed about the risks associated with their medication:

*It was a big shock because I didn’t really know about anything until the chemist was speaking to me about it […] I didn’t have a clue*.

(M, 65)

In addition to having low levels of overdose awareness, all patients reported that they perceived themselves as being at low risk of overdose: ‘*The chance of me having an overdose are very, very unlikely*’ (M, 74). Perceiving themselves as being at low risk of opioid overdose also impacted on how suited patients thought they were to the intervention:

*I really don’t feel that I need this […] I personally don’t think that I would even accidentally overdose*.

(W, 67)

Several patients differentiated themselves from people who used non-prescription drugs. This was particularly apparent when discussing risk of overdose and suitability of the intervention. Patients only discussed risk related to non-prescription drug use:

*When he said it was to do with drug overuse or drug abuse, naturally you think about drug addicts. […] I thought, well I’m not a drug addict, I don’t inject myself, I don’t abuse my medication*.

(M, 51)

Following the intervention, all patients acknowledged that, no matter how stringent they were in terms of dosage and consumption, they were still at risk of overdose to some extent:

*I wouldn’t have thought that I was at risk of having an overdose, but I am. And I now accept that to a degree*.

(M, 74)

#### 3.2.2. Theme Two: Experiences and Perceptions of Support

Patient satisfaction was closely related to their perceptions of intervention content and delivery by community pharmacists. For example, patients appreciated the time pharmacists spent explaining the intervention to them:

*He [the community pharmacist] was very, very good. I mean sometimes I’m hard of hearing and he took his time to explain things because sometimes I’ve to watch people’s lips. He was very informative and straightforward with it. It was very, very relaxed, it really was. And I was under no pressure whatsoever*.

(M, 51)

Patients also felt supported by their community pharmacies more generally. During the COVID-19 pandemic, GP appointments were difficult to access, and some patients accessed pain management advice they would have previously received from their GP with guidance from their community pharmacist. Patients perceived community pharmacies as being increasingly important in attending to their health needs during the pandemic and valued them more than they had before:

*I find that the relationship with the pharmacy is much more important to a degree than it is with the GP surgery, particularly so since COVID-19 because of the lockdown procedures*.

(M, 74)

In addition to considering support from pharmacy staff, some patients also discussed the importance of being supported by family members during the intervention. Two patients had memory problems and having someone present with them whilst receiving the intervention was essential so that opioid safety information could be remembered:

*Can I just say something? I’ve got [redacted to retain anonymity] disorder and my wife is here with me. I’ve got a memory like a sieve and a lot of the time it’s my wife that knows the answer*.

(M, 47)

Whilst some patients were supported by a family member during the intervention, others attended alone and shared information with their family and carers after. Information gained through the intervention was not only a comfort for some patients, but it could also provide comfort for family members, whether or not they attended the intervention:

*It doesn’t just give me peace of mind, it gives my family peace of mind as well*.

(M, 51)

#### 3.2.3. Theme Three: Perceptions of Intervention and Participation

Patients generally appreciated the pharmacist taking time to talk with them about their medications, and that they were not rushed out of the consultation room: ‘*He was really good, talked to me in a lot of detail*’ (W, 57). Patients reported that pharmacists had comprehensively explained the intervention, used an appropriate tone, and provided an opportunity for questions:

*I would give it ten [out of ten] because he genuinely took his time to sit and explain things to me. And it was such a relaxed atmosphere*.

(M, 51)

However, not all patients reported that they received this level of detail, with some inconsistency reported across pharmacies. For example, some patients were in the pharmacy for a long time: ‘*An hour we were in for*’ (M, 65), but others were: ‘*Less than five minutes*’ (W, 67). The variance in delivery of intervention was reflected in patient reported satisfaction:

*She [community pharmacist] never really told us much about it […] Just spray it up your nose and that was it*.

(W, 67)

No patients reported or implied that they did not enjoy or regretted participation in the intervention, even when they had suggestions for improvement. For example: ‘*I mean I’m a 67 year-old woman, so I understand, it’s nae [not] my age group you need to target*’ (W, 67). This participant felt that the intervention would be most suited to younger people, as she considered them as having less understanding of the risks related to high strength opioids.

In summary, patients generally had low overdose awareness and low risk perceptions related to their opioid use. Low levels of perceived risk contributed to those interviewed initially feeling that the intervention should be provided to people who use non-prescription drugs and not them, but after the intervention, all patients acknowledged that they were experiencing some levels of risk related to their opioids. Whilst experiences of the intervention varied, all patients had positive attitudes towards the intervention.

### 3.3. Pharmacist Interview Findings

Four pharmacists participated in interviews and three themes were developed from the analysis: healthcare system and naloxone perspectives; patients’ risk awareness and suitability for intervention; and perceptions of intervention content and delivering the intervention. Overall, pharmacists reported that the intervention provided comprehensive and appropriate information to CNCP patients to reduce risk of opioid overdose and addressed a gap in patients’ overdose awareness.

#### 3.3.1. Theme One: Healthcare System and Naloxone Perspectives

Pharmacists discussed their perceptions of the intervention relative to NHS processes and systems, and experiences with opioids and naloxone. When discussing the intervention, and wider experiences of opioid dispensing, systemic barriers to improving patient opioid safety were highlighted, including breakdowns in communication between patients and GPs:

*They [doctors] should be telling their patients about the risks and the side effects, instead of just prescribing them [opioids]. Because I feel that’s what they do a lot of the time. And then they [patients] will come in here and I’ll explain the risks, and they will be like, “Oh the doctor never said.” And some of them are actually a bit wary about going onto the opioids once they hear the risks because you know it’s, it can’t, well, obviously it’s quite dangerous and it causes a lot of death*.

(Pharmacist one)

Challenges in communication between GPs and pharmacies were also outlined. For example, pharmacy staff noted that changes to medication prescribed by GPs were not easily viewed on the pharmacy systems. A lack of cross-professional communication was also highlighted as preventing more comprehensive and fluid discussions of overdose risk for CNCP patients prescribed opioids:

*There is not much communication between the GPs and pharmacies regarding increasing or decreasing dose. It’s just maybe by chance if you are looking at their file and you see that, “oh you know, that’s different to what they used to be on.” Yeah, it’s not, it’s not quite as obvious as you might hope*.

(Pharmacist four)

Communication with patients was also discussed, particularly regarding how best to convey opioid overdose risk messages. Even within pharmacies in areas with high levels of prescription opioids, discussion of opioid-related risk with CNCP patients was uncommon:

*I would say that it was one of the more trickier subjects. It’s not really something we would discuss with people that were on opioids that weren’t like substance misuse. So, like we do that for substance misuse, but not for people who take prescribed opioid drugs*.

(Pharmacist two)

Related to discussions of opioid overdose risk, pharmacists discussed their perceptions of naloxone, which they described as a valuable and essential harm reduction intervention: ‘*I think if you are a pharmacy, it should be something that is a necessity and not an option*’ (Pharmacist one). When considering administration, most pharmacists preferred Nyxoid^®^ (intranasal spray) which was identified as increasing usability and acceptability of naloxone: ‘*Giving someone a nasal spray is much more acceptable to me than giving an injection to someone I don’t know*’ (Pharmacist three).

#### 3.3.2. Theme Two: Patients’ Risk Awareness and Suitability for the Intervention

Pharmacists outlined that developing patients’ understandings of overdose risk and safe consumption behaviours was an essential component of the intervention. In general, their view was that patients prescribed opioids for CNCP had a lack of knowledge of factors that increased overdose risk:

*So, the patients that we did contact, they received the information very well. They weren’t that knowledgeable, in fact, [that they were] at a higher risk, compared to others, of opioid overdose*.

(Pharmacist two)

According to the pharmacists interviewed, it was not unusual for patients to be uninformed about basic information related to their prescribed opioids and they noted that it frequently fell to pharmacists to bridge this gap:

*A lot of the recruits that I managed to get were on tramadol. And at least two if not three of those recruits were very surprised to learn that tramadol was morphine-based. So that was a bit of a shock to those recruits*.

(Pharmacist three)

Related to perceptions of risk was perceptions of suitability for the intervention. Pharmacists had varying success with the recruitment process, and one pharmacist had no experience of recruiting (representative of non-participating pharmacists). Pharmacists discussed how they approached patients, and why some groups were excluded. Whilst study criteria did not stipulate that delivery patients (patients who received their prescriptions by delivery rather than by in-person visits to the pharmacy) should be excluded from the study, pharmacists found it easier to recruit patients who came into the pharmacy:

*Quite a large number of patients who I identified as being eligible for the study, were, in fact, delivery patients, and so we didn’t really have a lot of face-to-face contact with them. And I think that I probably swayed towards excluding those from the study because I didn’t feel like talking to them over the phone would have allowed proper engagement and proper understanding to be achieved*.

(Pharmacist three)

#### 3.3.3. Theme Three: Perceptions of Intervention Content and Delivering the Intervention

Pharmacies participated under conditions of staff absence and shortages, time pressures, and the COVID-19 pandemic; however, all pharmacies perceived the intervention as being valuable with regards to reducing risk for patients:

*It was, all in all, a very positive experience. And just feeling that I was helping them as well for something potentially if it was to go wrong in the future. I had given them that little bit of help*.

(Pharmacist four)

Pharmacies were reimbursed from study funds for the naloxone they supplied and received a payment for each patient recruited into the study, and for each patient who received the intervention from the study funds (in Scotland naloxone is provided at no cost to patients at risk of overdose). Due to their investment of time and resources in the intervention and study, it was essential that staff felt their participation was fairly compensated. An important consideration for the feasibility of the intervention was the value for money that it provided. Pharmacy staff considered how much time would need to be invested in delivering the intervention, which they compared with other services to gauge acceptable remuneration:

*The EHC—[the emergency hormonal contraception provision] contract—does attract a fee I think of £25 or £30, or whatever it is per consultation. So, probably given the time it takes to have a conversation about their prescription opioids, that would probably be a fair remuneration for a patient*.

(Pharmacist three)

The mode of intervention delivery was also discussed. While the study was designed so that the intervention could be delivered either in-person at the pharmacy or remotely using video conferencing software, all patients chose to attend in-person. Interviewed pharmacists identified that they would not have the time to learn how to use the ‘Near Me’ software that was required: ‘*The NHS board just isn’t set up on a community level, at least, for ‘Near Me’ consultations. I can’t even fathom how we would go about setting that up on a practical level*’ (Pharmacist three).

Pharmacists delivered the intervention supported by materials which were created by the research team (see Methods section ‘intervention’) which pharmacists described as comprehensive: ‘*It covered everything that I was maybe expecting it to cover and more*’ (Pharmacist four), and: ‘*The materials that you sent through were very direct and they were very succinct’* (Pharmacist three). The intervention content was designed to be comprehensive yet concise due to limitations of time, and so that patients were not overwhelmed with information.

In addition to strengths of the intervention, pharmacists identified barriers, including staff changes, time constraints, general pressures of working in a community pharmacy, and changes during the pandemic. Pharmacists also proposed how the intervention could be improved:

*The intervention went perfectly well, and they were able to understand, but I think having them hold it [the demonstration intranasal spray] in their hands and being able to practice what it feels like […] I think that might have been beneficial*.

(Pharmacist four)

In summary, pharmacists delivered essential opioid overdose awareness knowledge to CNCP patients who were prescribed high-strength opioids. Pharmacists thought the intervention materials were comprehensive but indicated barriers in the potential reach of intervention due to lack of confidence in using video conferencing methods.

## 4. Discussion

This study illustrates the experiences of a sample of patients prescribed high-strength opioids for CNCP, and community pharmacists in receiving and providing a customised overdose prevention intervention in one NHS board in Scotland.

Two key findings outline that, prior to delivery of the intervention, patients had no or very low overdose awareness knowledge and, related to this, patients considered themselves as being at a very low risk of opioid overdose. Generally, patients did not have knowledge of overdose signs and symptoms, and perceived risk only related to non-prescription drug use. Patients identified that, following the intervention, they had a better understanding of opioid-related risks and how they could minimise these. They also reported feeling better educated in overdose awareness and naloxone administration. Low awareness of opioid overdose signs and symptoms in a CNCP population has also been identified by Nielsen et al. [38] who proposed that prescribers should be required to discuss risk of overdose with their patients. Nury et al. [11] also identified that informing the CNCP population about opioid-related harms is increasingly important given that the trend to prescribe opioids continues to rise in many countries. Aligned with Nielsen et al. (2018) and related to the current study’s findings about breakdowns in how risk of high strength opioids is communicated to patients, future related research should consider also engaging with prescribers to gain further insight into how opioid-related risk and harms are discussed with patients. Engaging with prescribers may also provide the opportunity for informal patient feedback.

Informed by clinical expertise and the British National Formulary [33], this study included ten medications in the definition of ‘strong opioids’. Whilst these drugs all have the potential to cause overdose, they are not equally reflected in Scottish drug-related deaths. Of the 1119 opiate/opioid-related DRD in 2021, methadone was implicated in 57%, (dihydro)codeine in 17%, and buprenorphine in 11% [39], often in combination with other non-prescribable substances. It should be noted that, in Scotland, methadone and buprenorphine are more commonly prescribed to treat opioid (heroin) use disorder than as analgesics. Excluding DRD attributed to multiple substances, in that year just ten deaths were attribute to tramadol alone, and six to oxycodone alone [40]. To ensure cost effectiveness, future interventions may wish to refine inclusion criteria based on the types of medications and prescribing patterns (e.g., polypharmacy, high dose, and extended-release drugs) known to be associated with harm, informed by surveillance data.

Initially, most patients in the current study were unsure if they were suited to the intervention, and this was related to preconceptions that naloxone was only for people who use non-prescription drugs. Such hesitancy around naloxone acceptance in a CNCP population was also outlined by Dassieu et al. [41] who suggested that, whilst pharmacists tended to think of naloxone as an important tool to reverse overdose, patients who experienced opioid-related stigma were hesitant to accept naloxone due to concerns they would be labelled as being opioid dependent. Hesitancy for CNCP patients to accept naloxone was also identified by Mueller et al. [42]. To facilitate acceptance of naloxone, Mueller et al. (2017) found that describing naloxone as a safety measure to be used in emergency situations resonated with patients who did not consider themselves at risk of overdose, given it communicated the risk related to the medication without judgement. Lack of patient understanding of overdose risk was similarly noted as a key barrier to pharmacist delivery of opioid harm reduction interventions in community settings by Nichols et al. [43]. Future harm reduction interventions for the CNCP population may therefore benefit from communicating naloxone as an intervention to increase safety, rather than mitigate overdose risk, as this patient group generally do not perceive themselves as being at risk.

Patients were generally very satisfied with the delivery of the intervention by community pharmacists, saying that they felt listened to about their pain and that pharmacists communicated sensitive information, such as overdose risk, without causing distress. Mirroring current findings, Tabeefar et al. [44] also outlined that community pharmacists were knowledgeable and empathetic towards patients with CNCP. Aligned with Matheson et al. [26] and Moustaqim-Barrette et al. [27], the current study has highlighted the utility of engaging with community pharmacies to increase accessibility and availability of naloxone in the community.

This study has also outlined important learning related to implementation challenges within community pharmacies. The need to work strictly within a study protocol that had ethical and NHS governance approvals meant that the intervention could not be readily and opportunistically flexed––for example, if someone narrowly missed being eligible but was keen to participate. Similarly, a conversation with another non-participating pharmacist (not formally interviewed) identified that the complexity of the current study’s research process was a barrier to both community pharmacy and patient participation. The pharmacists proposed that streamlining the consent and recruitment processes would make the intervention more ‘pharmacy friendly’. We would therefore suggest that these processes should be revised if the intervention was piloted as a service improvement exercise rather than a research project.

Whilst it was feasible for pharmacists to deliver a customised overdose prevention intervention to CNCP patients in the community, the reach of the intervention in practice was limited to patients who visited the pharmacy in-person, meaning patients who received delivery prescriptions were not offered it. To improve accessibility and reach to such patients, creating additional support for remote delivery is important. However, delivering the intervention remotely is reliant on the technological skills, confidence, and access to equipment on the part of patients as well as pharmacists. In their exploration of e-health literacy in pharmacy staff in Scotland, MacLure, and Stewart [45] identified that, whilst pharmacy staff may have access to software, many self-reported a lack of confidence and digital literacy in their information technology systems. Authors outlined the requirement for investment in pharmacy staff training to support e-health literacy, in addition to the provision of e-health technology [45].

The study was limited by difficulties in recruiting both community pharmacies and patients to deliver the intervention to. The limited uptake in this feasibility study is likely to be related to the study being conducted during COVID-19. There were significant pressures on community pharmacies during this time, and delivery of essential services were the focus of most. Pharmacies were reimbursed for their time, naloxone, and all study resources, so cost was not perceived to be a barrier to uptake. Limited uptake by pharmacies reduced the population of CNCP patients the study was able to reach. This is a key weakness of the study which was designed to be delivered in more settings to many more patients, and would have engaged a more representative sample of pharmacists and patients. The intervention was at no cost to the patient and was fully funded, so cost was not considered as a barrier. However, as outlined in the findings, many patients did not perceive themselves as being at risk and did not therefore see themselves as being eligible for a harm reduction intervention. Patient risk perceptions could thus be considered a barrier for recruitment. Nonetheless, the limited uptake provided important learning that will inform changes to the intervention design before it is proposed as trial stage. While the small sample limits generalisability to similar patient groups, participants had a similar age, comorbidity and polypharmacy profile to high-dose opioid recipients elsewhere in Scotland [6], and findings do offer a unique insight into the potential of this intervention in Scotland and more widely. Notwithstanding the challenges encountered, the study is unique in Europe in targeting the CNCP population with an overdose prevention intervention that includes opioid risk education, naloxone training and provision. Learning from the current study includes knowledge of: the time needed to collect data; the ability of pharmacists to recruit patients; pharmacist views on the mode; and supporting conditions for the intervention and follow up rates, all of which are important outputs of a feasibility study that can, in turn, help design future randomised control trials [46].

While opioids that can be prescribed are implicated in a substantial number of DRDs in Scotland, this population is underserved. Despite a small sample due to contextual challenges related to the COVID-19 pandemic, this overdose prevention intervention tailored for CNCP patients prescribed high-strength opioids improved patient overdose awareness knowledge, increased patient understanding of opioid-related risk, and highlighted the value of naloxone as a safety measure in the CNCP population. As part of a comprehensive strategy to reduce opioid-related harms, the safety of CNCP patients prescribed high-strength opioids that are implicated in medication-related morbidity and mortality must be urgently considered [13].

## Figures and Tables

**Table 1:** Medications taken by patients.

Drug	Number	Proportion
Opioid	12	100%
Analgesic (paracetamol/ibuprofen)	7	58%
Gabapentin/pregabalin	5	42%
Antidepressant (e.g., amitriptyline)	5	42%
Antihistamine (e.g., promethazine)	2	17%
Non-steroidal anti-inflammatory (e.g., diclofenac)	2	17%
Antacid/proton pump inhibitor (e.g., omeprazole)	2	17%

**Table 2:** Prescription Opioid Misuse Index (POMI) responses.

POMI Risk Factors	Number	Proportion
Use pain medication more often than is prescribed.	3	25%
Need early refills for pain medication.	3	25%
Gone to a different doctor or an A&E unit to try to get more pain medication.	1	8%
Take a higher dose than prescribed	0	0%
Feel high or get a buzz after using pain medication	0	0%
Take pain medication because upset, or to relieve or cope with problems other than pain.	0	0%

**Table 3:** Patient comorbidities.

Condition	Diagnosed But Not Being Treated		Diagnosed and Being Treated	
Cardiovascular	1	8%	4	33%
Respiratory	2	17%	6	50%
Renal	1	8%	0	0%
Sleep apnoea	0	0%	2	17%
Liver	2	17%	0	0%
Alcohol/drug problem	0	0%	0	0%

**Table 4:** Responses to follow-up questionnaire about views on the intervention.

	Patient Identifier (Gender and Age)		
	M 74	M 51	M 72
Please tell us what you thought about the information you received			
How helpful did you find the information overall?	Very	Very	Extremely
How easy to understand was the printed/online information?	Extremely	Very	Extremely
How easy to understand was the information provided by the community pharmacist?	Very	Extremely	Extremely
The information was relevant to me	Extremely	Moderately	A little
The information will be relevant to other people who are prescribed opioids.	Extremely	Extremely	Very
To what extent do you agree or disagree with the following statements?			
I learned new things about the risk of prescription opioid overdose.	Agree	Agree	Neither agree nor disagree
I learned how to reduce my risk of having an overdose.	Neither agree nor disagree	Strongly agree	Neither agree nor disagree
I learned what steps someone around me should take if they think I am having an overdose.	Strongly agree	Strongly agree	Strongly agree
I am confident talking to people I live with about what they should do if they think I am having an overdose.	Strongly agree	Strongly agree	Does not apply to me
The information and naloxone training caused me to feel upset.	Strongly disagree	Strongly disagree	Strongly disagree
The information and naloxone training caused someone I live with to feel upset.	Disagree	Strongly disagree	Does not apply to me
I would recommend the information and naloxone training to other people who are prescribed opioids.	Strongly agree	Strongly agree	Agree

## Data Availability

The data presented in this study are available on request from the corresponding author.

## Supplementary Materials

The following supporting information can be downloaded at: https://www.mdpi.com/article/10.3390/pharmacy11030088/s1, Supplementary File S1: Patient interview topic guide; Supplementary File S2: Pharmacy staff interview topic guide.
