# Supplementary material for: Feasibility and Acceptability of an Overdose Prevention Intervention Delivered by Community Pharmacists for Patients Prescribed Opioids for Chronic Non-Cancer Pain
Source: Pharmacy (Basel). 2023 May 22;11(3):88. doi: 10.3390/pharmacy11030088 (PMC10204494; doi:10.3390/pharmacy11030088)
Supplement: Supplementary file 1 [file pharmacy-11-00088-s001.zip › pharmacy-2348657-supplementary.pdf]

Supplementary material A—Patient interview topic guide

Patient Topic Guide: to explore patients' perceptions of the feasibility and acceptability of the THN intervention.

Introductory script

Hello and thank you for talking to me today. In this interview it would be great to hear about your experiences of the opioid safety project and what you thought of it. Since I'm asking your opinion, there is no right or wrong answer - I'm simply interested in hearing what you think. I'll change identifiable information, such as names and places, so others won't be able to tell what you've said, and all information you tell me will be kept confidential unless I'm worried you might hurt yourself or be at serious risk. If this is the case, I'll need to pass on what you've told me to my supervisor, and appropriate support will be provided. The interview will last around 45 minutes, but you can skip any question you don't want to answer. Your participation is voluntary, and you are free to withdraw at any time. If you have any questions, our contact details are on the debrief sheet. Does all this sound okay? If so, are you happy to get started? Before we begin, is there anything you would like to ask me?

Invitation to participate

I'd like to hear about your experience of being asked to take part in the project. Can you tell me a bit about what it was like being invited to take part?

Prompts

a. How did you feel when you were asked to take part?
b. What were you told about the intervention before taking part?
c. Did you have any questions/ concerns before taking part? If so, were they answered fully?

Intervention materials

I'd like to ask you to think back to when you received the intervention. Did you choose to receive the intervention face to face in the pharmacy, or via 'Near me'?

Prompts

a. Did anyone attend the training with you? If so, who joined?
b. What did you think of the information provided to you by the pharmacist, both written and verbal?
c. If anything, what else might have been useful to include in these materials?

Naloxone training

Everyone has a different experience of naloxone training, so it would be great to know what you thought about it. If you were to rate your training out of 10 (1 being poor and 10 being brilliant) what score would you give it and why?

Prompts

a. What, if anything, did you find most useful about the training?
b. What, if anything, did you find least useful about the training?
c. How confident did you feel about using naloxone after your training?

d. What suggestions, if any, do you have on how we could improve training?

Naloxone use

After the training, were there any times when naloxone was administered? If so, can you tell me more about this? This can be an example of a situation where you received or provided naloxone.

Prompts

a. Can you tell me about circumstances where the naloxone training helped you or others? (This doesn't need to be just in an overdose situation, for example, it could be using your knowledge from the intervention to answer a question for a friend)
b. In what situations, if at all, have you consulted information from the materials, for example, the information sheet?

Perceptions of health and medication

- Has participation impacted your understanding of your pain? How?
- has participation impacted your understanding of the medication you take for your pain? How?
- has participation impacted your understanding of overdose risk, and risk factors? How?

- Have you sought advice on your managing your pain, medication or addressing overdose risks?
    a. If so, who did you discuss this with (GP/ pain service/ voluntary sector etc)?
    b. What, if anything, changed because of this?

Improving the intervention

As we finish, it would be great to hear about any suggestions you might have to improve the intervention.

Thank you for taking part in this interview. Your responses have been recorded and will provide useful information about naloxone intervention for people prescribed opioids for non-cancer pain. Before we leave, are there any other questions you would like to ask me, or any comments you would like to add?

Supplementary material B—Pharmacist interview topic guide

Pharmacist topic guide: to explore pharmacists' perceptions of the feasibility and acceptability of the THN intervention.

Introductory script

Hello and thank you for talking to me today. We are looking to interview as many staff as possible who are participating in the POOR 2 trial to understand and learn from experiences so far. There is no right or wrong answer - I'm just interested in hearing what you think. I'll change identifiable information, such as names and places, so others won't be able to identify you from what you've said. All information you tell me will be kept confidential. The interview will last around 15-20 minutes, and you can take a break, skip any question, or decide to leave without any reason at any time. Your participation is voluntary, and you are free to withdraw at any time. There are contact details on the debrief sheet where you can contact us if you have any questions after the interview. Does this sound okay? If so, are you happy to get started? Before we begin, is there anything else you would like to ask me?

Background

- Could you briefly introduce yourself and your role in the community pharmacy?

Prompts

a. How long have you been in this role?
b. How equipped did you feel before this research project to discuss opioid related risks and naloxone?
c. How, if at all, has this project developed your knowledge of opioid overdose risk for people prescribed opioids for chronic non-cancer pain?

Knowledge and Training

- To start it would be good for you to tell me about your experience of naloxone training for the study.
- Had you previously been delivering naloxone e.g. to people prescribed methadone or buprenorphine or accessing needle exchange?
- How prepared did you feel to talk to patients about naloxone and risk of overdose following SDF training and reading project materials?
- Did you find the online Teams session was beneficial?

Prompts

a. How confident did you feel providing advice and support to patients?
b. If anything, what else would you have liked to be included or know more about in the training?
c. Do you think that the available training covered identify and responding to overdose risk in people prescribed opioid analgesia sufficiently?

Recruitment

- I'd like to hear about your experience of recruitment. What was it like to find eligible participants and invite them to participate?

Prompts

a. How easy or difficult did you find recruitment?
b. How did you approach discussing the project with patients when talking with them in the pharmacy?
c. What went well when recruiting patients? (e.g., did the pharmacy have records of eligible patients, how receptive were patients to discussing overdose risk?)
d. Did you have any experiences which didn't go as well?
e. What if anything, might have made recruitment easier for you?

Delivery

- When delivering the intervention (i.e., taking patients through the materials and providing naloxone and training on its use), there was the choice of face to face or 'Near me'.
- Does your pharmacy have "near me" available?
- In general, what was the preferred mode of delivery?
- How would useful were the support materials provided?

Prompts

a. Who determined the preferred mode of delivery, the pharmacy or the patient? i.e. did you offer both options?
b. If both were available, did you have a preferred mode of delivery? If so, why?
c. Do you think the modes of delivery were appropriate for the intervention? Please provide some information on why you think they were/ weren't.
d. Do you think COVID-19 impacted on the ability to deliver the intervention?
e. Are there any changes you would make to the support materials provided?

Implementing the trial

- What was your experience of delivering the intervention to patients?

Prompts

a. What worked well?
b. What didn't work well?

Cost models

- When you compare against other services delivered by community pharmacy, what do you think an appropriate fee for the time and resources needed to deliverer this intervention would be?

Value/ feasibility

- Overall, how do you think this project fits with the role, processes and pressures in community pharmacy? (In and out of COVID?)
- If we were to implement this intervention in other areas in the future, what, if anything, could we do to improve it?

- From your experience of participation, what value or importance, if any, does this intervention have for patients who are prescribed prescription opiates for non-cancer pain?
- Are you aware of any difference the project made to patients and their opioid overdose risk? (e.g. did you note any changes to prescriptions, did patients report any discussions with their prescriber as a result or changes to their medication type or dose, did patients ask questions about the medication or risk factors?)
- Are you aware of any patients who have had to use their naloxone? If yes do are you able to give an overview of this?
- Did patients express any concerns as a result of their participation? How did you / other pharmacy staff address these.

Improving the intervention

Just to finish, it would be great to hear about any suggestions you might have to improve the intervention which you haven't already mentioned.

Thank you very much for taking part in this interview. Your responses have been recorded and will provide useful information about naloxone intervention for people prescribed opioids for non-cancer pain. Before we leave, are there any other questions you would like to ask me or any comments you would like to add?
